# An Enzyme-Linked Immunosorbent Spot Assay Measuring Borrelia burgdorferi B31-Specific Interferon Gamma-Secreting T Cells Cannot Discriminate Active Lyme Neuroborreliosis from Past Lyme Borreliosis: a Prospective Study in the Netherlands

**DOI:** 10.1128/JCM.01695-17

**Published:** 2018-03-26

**Authors:** T. van Gorkom, S. U. C. Sankatsing, W. Voet, D. M. Ismail, R. H. Muilwijk, M. Salomons, B. J. M. Vlaminckx, A. W. J. Bossink, D. W. Notermans, J. J. M. Bouwman, K. Kremer, S. F. T. Thijsen

**Affiliations:** a Department of Medical Microbiology and Immunology, Diakonessenhuis Hospital, Utrecht, the Netherlands; b Department of Internal Medicine, Diakonessenhuis Hospital, Utrecht, the Netherlands; c Department of Neurology, Diakonessenhuis Hospital, Utrecht, the Netherlands; e Department of Pulmonology, Diakonessenhuis Hospital, Utrecht, the Netherlands; d Department of Medical Microbiology and Immunology, St. Antonius Hospital, Nieuwegein, the Netherlands; f Laboratory for Infectious Diseases and laboratory Surveillance, Centre for Infectious Diseases Control, National Institute for Public Health and the Environment (RIVM), Bilthoven, the Netherlands; Medical College of Wisconsin

**Keywords:** Borrelia burgdorferi, ELISpot, Lyme borreliosis, Lyme neuroborreliosis, T-cell activation, active disease, antibodies, cytokines, diagnostics, interferon gamma

## Abstract

Two-tier serology testing is most frequently used for the diagnosis of Lyme borreliosis (LB); however, a positive result is no proof of active disease. To establish a diagnosis of active LB, better diagnostics are needed. Tests investigating the cellular immune system are available, but studies evaluating the utility of these tests on well-defined patient populations are lacking. Therefore, we investigated the utility of an enzyme-linked immunosorbent spot (ELISpot) assay to diagnose active Lyme neuroborreliosis. Peripheral blood mononuclear cells (PBMCs) of various study groups were stimulated by using Borrelia burgdorferi strain B31 and various recombinant antigens, and subsequently, the number of Borrelia-specific interferon gamma (IFN-γ)-secreting T cells was measured. We included 33 active and 37 treated Lyme neuroborreliosis patients, 28 healthy individuals treated for an early manifestation of LB in the past, and 145 untreated healthy individuals. The median numbers of B. burgdorferi B31-specific IFN-γ-secreting T cells/2.5 × 10^5^ PBMCs did not differ between active Lyme neuroborreliosis patients (6.0; interquartile range [IQR], 0.5 to 14.0), treated Lyme neuroborreliosis patients (4.5; IQR, 2.0 to 18.6), and treated healthy individuals (7.4; IQR, 2.3 to 14.9) (*P* = 1.000); however, the median number of B. burgdorferi B31-specific IFN-γ-secreting T cells/2.5 × 10^5^ PBMCs among untreated healthy individuals was lower (2.0; IQR, 0.5 to 3.9) (*P* ≤ 0.016). We conclude that the Borrelia ELISpot assay, measuring the number of B. burgdorferi B31-specific IFN-γ-secreting T cells/2.5 × 10^5^ PBMCs, correlates with exposure to the Borrelia bacterium but cannot be used for the diagnosis of active Lyme neuroborreliosis.

## INTRODUCTION

In the Netherlands, Lyme borreliosis (LB) poses a considerable threat to human health. A study among general practitioners (GPs) found a threefold increase of patients reporting tick bites and diagnoses of erythema migrans (EM), an early, localized skin rash, in the period between 1994 and 2009 (1). Between 2009 and 2014, the incidence of reported tick bites ranged between 488 and 564 consultations per 100,000 inhabitants and the number of GP-reported diagnoses of EM ranged between 134 and 140 per 100,000 inhabitants (2). The true incidence rate is probably higher, since only a small part of the population consults a GP after a tick bite (3). Increased incidences of LB have also been reported in several other European countries as well as in the United States (4–6).

Diagnosis of active LB can be difficult in the absence of a “gold standard” test, such as PCR or culture. Exceptions are an EM, which is a clinical diagnosis, and acrodermatitis chronica atrophicans (ACA) or Lyme arthritis, which can be supported by PCR and/or culture. For Lyme neuroborreliosis, culture and PCR are too insensitive to be useful in a routine clinical setting (7–10). The diagnosis of Lyme neuroborreliosis is based on clinical symptoms and needs to be supported by laboratory tests. The most frequently found clinical symptoms of Lyme neuroborreliosis are (lymphocytic) meningoradiculitis and paresis (11); however, symptoms can be nonspecific, which often complicates the diagnosis. Confirmation of Lyme neuroborreliosis through laboratory testing consists of the detection of Borrelia-specific antibodies in cerebrospinal fluid (CSF) and an elevated number of mononuclear cells in CSF, otherwise known as pleocytosis (≥5 leukocytes/μl) (11). Unfortunately, studies using well-characterized and unbiased patient groups are rare and the sensitivity and specificity of the various tests can vary extensively (12).

The presence of intrathecally produced Borrelia-specific antibodies can indicate active Lyme neuroborreliosis but also the persistence of Borrelia-specific antibodies years after an asymptomatic or treated infection (13). The absence of Borrelia-specific antibodies, on the other hand, does not rule out an active infection and can be explained by the (low) sensitivity of the test used and the time it takes for the body to produce detectable levels of Borrelia-specific antibodies after an infection (14). As early and correct diagnosis of Lyme neuroborreliosis is essential for adequate treatment with antibiotics (15–17), better diagnostic tools are warranted. The diagnostic shortcomings underline the need for new diagnostic tests that can distinguish between active disease and a previous, yet cleared, infection or that can aid in the diagnosis for those cases for which the current diagnostics are insufficient. In this study, active Lyme neuroborreliosis patients were used as a proxy for active disease.

In recent years, assays that focus on the cellular immune response for the diagnosis of LB have become available. The cellular immune response against Borrelia is characterized by a strong Th1 response, in which Borrelia activates Th1-like cytokines such as interferon gamma (IFN-γ) (18–20). Elevated amounts of Th1-specific IFN-γ in blood, synovial fluid, and CSF of LB patients have been found in various studies (21–25). However, compared to serology, T-cell assays were less sensitive and specific, and in general these assays were not well standardized (10, 26). Despite the lack of published studies on clinically validated cellular assays, various laboratories offer these assays for the diagnosis of LB. These assays include the enzyme-linked immunosorbent spot (ELISpot) assay (27) and the lymphocyte transformation test (LTT) (28). Therefore, the clinical validation of these assays is urgently needed.

In this study, we performed the validation of a Borrelia ELISpot assay measuring the number of IFN-γ-producing T cells after stimulation with Borrelia burgdorferi B31. Information regarding previous tick bites, symptoms, and antibiotic treatment for LB was assessed by the completion of a Lyme-specific questionnaire and through consulting electronic patient files. We used a standardized assay on well-defined groups of both treated and untreated patients and healthy controls to investigate whether the number of Borrelia-specific T cells isolated from blood can be used as a marker for disease activity.

## MATERIALS AND METHODS

### Study population.

Whole-blood and serum samples were obtained from hospital patients diagnosed with active Lyme neuroborreliosis, hospital patients treated for Lyme neuroborreliosis in the past, and healthy individuals (all ≥18 years old). All hospital patients diagnosed with Lyme neuroborreliosis in Diakonessenhuis Hospital, Utrecht, and St. Antonius Hospital, Nieuwegein, the Netherlands, were eligible for inclusion in the study if they fulfilled at least two criteria for Lyme neuroborreliosis as defined by the European Federation of Neurological Societies (EFNS) (11). These criteria are (i) the presence of neurological symptoms suggestive of Lyme neuroborreliosis without other obvious explanations, (ii) CSF pleocytosis (≥5 leukocytes/μl), and (iii) Borrelia-specific intrathecal antibody production. If all three criteria were met, a case was categorized as a definite Lyme neuroborreliosis case, and if two criteria were met, a case was categorized as a possible Lyme neuroborreliosis case.

Hospital patients either were recently diagnosed with active Lyme neuroborreliosis or had been treated previously for Lyme neuroborreliosis. Active Lyme neuroborreliosis patients were recruited from December 2010 to December 2016 and were included if blood was drawn within 2 months after the start of antibiotic therapy. In addition, active Lyme neuroborreliosis patients could also be included as treated Lyme neuroborreliosis patients. To make sure that enough time had passed between both inclusions, at least 1 year should have passed after they had finished treatment for their Lyme neuroborreliosis disease episode. Treated Lyme neuroborreliosis patients, who had been diagnosed between February 2003 and September 2014, were enrolled from January 2011 to March 2015 and were included at least 4 months after completion of antibiotic therapy for Lyme neuroborreliosis.

Healthy individuals were recruited in the period between February 2013 and December 2015 from personnel of Diakonessenhuis Hospital, Utrecht, St. Antonius Hospital, Nieuwegein, and the National Institute for Public Health and the Environment (RIVM), Bilthoven, the Netherlands. Healthy individuals also included Boy Scout patrol leaders, owners of hunting dogs, and recreational runners. All were invited to participate if they pursued recreational activities in high-risk areas for tick bites. In addition, healthy individuals who had received antibiotic treatment for an early manifestation of LB in the past, as they had reported themselves in the Lyme-specific questionnaire, were analyzed as a separate group and are referred to as treated healthy individuals.

All hospital patients and healthy individuals were asked to complete a Lyme-specific questionnaire. This questionnaire included questions on tick bites, the presence of EM, antibiotic treatment for LB, and self-reported complaints at the moment of inclusion and during possible earlier episodes of LB. Information regarding the clinical symptoms, pleocytosis, and intrathecal antibody production during active disease of the Lyme neuroborreliosis patients was extracted from the hospital information system. Healthy individuals were recruited only if they reported no complaints at the time of the inclusion in the study. All study participants gave their informed consent. The regional Medical Research Ethics Committees United approved the study (Nieuwegein, the Netherlands; MEC-U: NL36407.100.11).

### Antibody detection in serum and serum-CSF pairs.

Borrelia-specific serum antibodies were detected using a two-tier serology protocol (29, 30). The first test used was the C6 enzyme-linked immunosorbent assay (ELISA) (Immunetics, Boston, MA, USA), which is based on a synthetic C6 peptide and is derived from a highly immunogenic part (invariable region 6) of the VlsE (variable major protein-like sequence, expressed) lipoprotein (31).

Equivocal and positive C6 ELISA results were confirmed by using the recomLine immunoglobulin M (IgM) and immunoglobulin G (IgG) immunoblot tests (Mikrogen GmbH, Neuried, Germany). The immunoblot strips detect antibodies against Borrelia burgdorferi sensu stricto, Borrelia afzelii, Borrelia garinii, Borrelia bavariensis, and Borrelia spielmanii by using different recombinant antigens (32). Each recombinant antigen has a certain value and will be counted when the intensity of the respective band is greater than or equal to the intensity of the cutoff band. The following antigens, with their respective scores, are used: p100 (IgM and IgG, 5 points each), VlsE (IgM and IgG, 5 points each), p58 (IgM and IgG, 4 points each), p41 (IgM and IgG, 1 point each), p39 (IgM, 4 points, and IgG, 5 points), OspA (IgM and IgG, 5 points each), OspC (IgM, 8 points, and IgG, 5 points), and p18 (IgM and IgG, 5 points each). Immunoblot results were recorded as negative (≤5 points), equivocal (6 points), or positive (≥7 points). Immunoblotting was performed according to the manufacturer's instructions, and the results were recorded with an automated recomScan system using the recomScan software (Mikrogen GmbH). The final immunoblot result was based on a combination of the results of both immunoglobulins: negative when both IgM and IgG were negative, equivocal when at least one of these was equivocal, and positive when at least one of these was positive. When immunoblot confirmation was performed, this result determined the final serology result, independent of an equivocal or positive C6 ELISA result.

Detection of intrathecally produced Borrelia-specific antibodies was done using the second-generation IDEIA Lyme neuroborreliosis test (Oxoid, Hampshire, United Kingdom) (33). Antibody index (AI) scores of ≥0.3 were considered positive. The final AI result was based on a combination of the results of both immunoglobulins: negative when the AIs of both IgM and IgG were negative, equivocal when at least one of these was equivocal, and positive when at least one of these was positive.

Both the C6 ELISA and the IDEIA Lyme neuroborreliosis test were performed according to the manufacturer's instructions using a Dynex DS2 automated ELISA instrument (Dynex Technologies), and results were analyzed with DS-Matrix software (Dynex Technologies).

### Borrelia ELISpot procedure.

The Borrelia ELISpot assay was performed on peripheral blood isolated from all study participants. The isolation of peripheral blood mononuclear cells (PBMCs) from whole-blood specimens (lithium heparin) drawn <8 h before testing was done through density gradient centrifugation (Hettich Rotanta 460 RS; rotor 5624) at room temperature for ∼15 min at 1,000 × *g* using Leucosep tubes (OxFord Immunotec Ltd., Abingdon, UK); however, for blood that was drawn between 8 and 32 h before testing, a T-cell Xtend (OxFord Immunotec Ltd.) step was performed following the procedure described by Bouwman et al. (34) prior to PBMC isolation. After centrifugation, the PBMC fraction was removed and washed twice. The first wash step was performed at room temperature for ∼7 min at 600 × *g*; the second wash step was also performed at room temperature for ∼7 min at 300 × *g*. Both wash steps were performed in 10 ml of fresh, prewarmed (37°C) RPMI medium (Life Technologies, Invitrogen, Bleiswijk, the Netherlands). If necessary, excess erythrocytes were removed between the first and second wash steps using human erythrocyte lysis buffer (0.010 M KHCO_3_, 0.0001 M EDTA, 0.150 M NH_4_Cl [pH 7.3 ± 0.1]). After addition of 5 ml of lysis buffer, the solution was incubated for 5 min at 2°C and subsequently centrifuged using the first wash step centrifugation program.

The final pellet was suspended in 1.1 ml of fresh, prewarmed (37°C) AIM-V medium (Life Technologies), and cells were counted using the AC.T diff 2 analyzer (Beckman Coulter, Woerden, the Netherlands). Cells were adjusted to 2.5 × 10^6^/ml and 100 μl of that concentration was added to a precoated polyvinylidene difluoride (PVDF) ELISpot^PRO^ well (Mabtech, Nacka Strand, Sweden). The negative control consisted of 50 μl of AIM-V medium, and as a positive control, 50 μl (0.1 μg/ml) of anti-human CD3 monoclonal antibody (MAb) CD3-2 (Mabtech) was used. To stimulate the cells, 50 μl of a whole-cell lysate (5 μg/ml), a peptide mix (5 μg/ml), and five recombinant antigens were tested (15 μg/ml). The whole-cell lysate tested was derived from B. burgdorferi strain B31 (Autoimmun Diagnostika GmbH, Straßberg, Germany). The peptide mix (an Osp mix) consisted of a pool of 9-mer to 11-mer peptides of OspA (B. burgdorferi, B. afzelii, and B. garinii), native OspC (B. afzelii), and recombinant p18 (Autoimmun Diagnostika GmbH). The five recombinant antigens used were (i) p18 B. burgdorferi sensu stricto PKa, (ii) p18 B. afzelii PKo, (iii) p18 B. garinii PBi, (iv) p39 B. afzelii PKo, and (v) p58 B. garinii PBi (Mikrogen GmbH), which are also part of the recomLine immunoblot test (Mikrogen GmbH). The number of different antigens tested depended on the yield of PBMCs. After 16 to 20 h at 37°C and 5% CO_2_, the wells were washed using phosphate-buffered saline (PBS; pH 7.2 ± 0.1) and incubated for 1 h at 2°C after addition of 50 μl of 7-B6-1–alkaline phosphatase (ALP) conjugate (Mabtech). The wells were washed again in PBS and incubated with 50 μl of 5-bromo-4-chloro-3′-indolylphosphate and nitroblue tetrazolium (BCIP/NBT) plus substrate (Mabtech) for ∼7 to 10 min at room temperature.

### Analysis of the Borrelia ELISpot assay results.

The number of Borrelia-specific IFN-γ-producing T cells, displayed as black spots, was measured with an ELISpot reader (Autoimmun Diagnostika GmbH), visually checked, and, if judged necessary, adjusted manually by two different operators in the EliSpot 6.0 software (Autoimmun Diagnostika GmbH). The spot size used was based on the expected spot size of an IFN-γ-producing T cell as determined by Feske et al. (35) and was set on −2.8 log (mm^2^). If there was a difference in the T-cell count between the two operators of ≥4 spots for a certain sample, or if they found any difference in spot count in the critical area (between 2 and 5 spots), then those samples were recounted by a third operator, whose result was leading.

To determine the actual spot count due to the stimulation of T cells by Borrelia, the number of spots in the negative-control well was subtracted from the number of spots in the antigen-stimulated well. The number of spots corresponds with the number of individual T cells producing IFN-γ after antigen stimulation. Different lot numbers of B. burgdorferi B31 lysate were used; however, they were derived from the same batch of B. burgdorferi B31 lysate. If a blood sample was tested with >1 lot number, or multiple times with an identical lot number, then the median spot count was calculated and used in the Borrelia ELISpot analysis.

### Data handling and statistical analysis.

For statistical analyses, the IBM SPSS software package (version 23) was used (IBM, Armonk, NY, USA). Dichotomous data were analyzed by using Pearson's chi-square test or Fisher's exact test. The *post hoc* tests consisted of two-group comparisons by using Pearson's chi-square test or Fisher's exact test using the Bonferroni correction. *P* values of <0.05 were interpreted as statistically significant. If the Bonferroni correction was applied, then a *P* value of 0.05/*k* (for which *k* is the number of different hypotheses) was interpreted as statistically significant. For statistical analyses, equivocal serology results were combined with positive serology results.

Quantitative, unrelated data comparing >2 groups were analyzed by using the Kruskal-Wallis test, and *post hoc* tests consisted of the Dunn-Bonferroni test. Quantitative, unrelated data comparing two groups were analyzed using the Mann-Whitney test. Correlations were calculated using Spearman's correlation coefficient (*r_s_*). Quantitative, related data comparing >2 tests were analyzed using Friedman's related-samples two-way analysis of variance test, and *post hoc* tests consisted of the Dunn-Bonferroni test. Quantitative, related data, comparing two tests, were analyzed using the Wilcoxon signed-rank test. For all analyses, *P* values of <0.05 were interpreted as statistically significant.

To determine the utility of the Borrelia ELISpot assay to diagnose active Lyme neuroborreliosis, a receiver operating characteristic (ROC) curve was created to calculate the area under the curve (AUC). Therefore, the number of Borrelia-specific IFN-γ-secreting T cells among active Lyme neuroborreliosis patients was compared with the number of Borrelia-specific IFN-γ-secreting T cells among the other three groups. Logistic regression was applied to investigate whether any additional risk factors could contribute to the diagnostic performance of the Borrelia ELISpot assay. The Hosmer-Lemeshow goodness-of-fit test was used to assess if the logistic regression model fit the data. The outcome of the model was binary: a case was either an active patient or a control. Figures were made with GraphPad Prism (version 5.04 for Windows; GraphPad Software, San Diego, CA, USA).

## RESULTS

### Study population. (i) Active Lyme neuroborreliosis patients.

Thirty-three active Lyme neuroborreliosis patients were included; their median age was 56.7 years (interquartile range [IQR], 44.8 to 64.4 years). They were included before, during, or shortly after antibiotic treatment started (median, 7.0 days after the start of antibiotic therapy; IQR, 3.0 to 12.5 days) (Table 1). Antibiotic therapy consisted of intravenous ceftriaxone for 14 or 30 days. Two patients switched to doxycycline because of an adverse reaction to ceftriaxone. One patient was given doxycycline from the start (21 days). The clinical symptoms among active Lyme neuroborreliosis patients mostly consisted of radicular disease (15/33 [45.5%]) and/or cranial nerve paresis (15/33 [45.5%]).

**TABLE 1:** Demographic and clinical characteristics of the four study groups^*a*^

Variable	Active Lyme NB patients^*b*^ (*n* = 33)	Treated Lyme NB patients^*b*^ (*n* = 37)	Treated healthy individuals (*n* = 28)	Untreated healthy individuals (*n* = 145)	*P* value	
					Overall	2-group^*c*^
No. of males (%)	22 (66.7)	19 (51.4)	13 (46.4)	55 (37.9)	0.020	0.003^*k*^
Median age, yrs (IQR)	56.7 (44.8–64.4)	59.3 (49.4–66.9)	52.7 (38.1–57.5)	41.0 (27.0–51.7)	<0.001	≤0.029^*l*^
Tick bite (%)	11 (45.8)^*d*^	27 (73.0)	26 (92.9)	87 (60.0)	0.001	≤0.041^*m*^
EM (*n*; %)	4 (16.7)^*d*^^,^^*e*^	9 (24.3)^*e*^	22 (78.6)^*h*^	4 (2.8)^*j*^	<0.001	≤0.015^*n*^
No. of positives in two-tier serology testing (%)	30 (90.9)	6 (16.7)^*g*^	5 (17.9)	18 (12.4)	<0.001	<0.001^*o*^
IgM (*n*; %)	16 (48.5)	3 (8.3)	2 (7.1)	1 (0.7)	<0.001	≤0.025^*p*^
IgG (*n*; %)	28 (84.8)	6 (16.7)	3 (10.7)	18 (12.4)	<0.001	<0.001^*q*^
Median time between end of AB and blood sampling, yrs (IQR)	NA	5.0 (2.5–7.3)	5 (2–7)^*i*^	NA	NA	0.563
Median time between start of AB and blood sampling, days (IQR)	7.0 (3.0–12.5)	NA	NA	NA	NA	NA
Self-reported complaints at inclusion	See Table 2^*f*^	23 (62.2%)	0	0	NA	NA

a EM, erythema migrans; AB, antibiotic treatment for Lyme borreliosis; IQR, interquartile range; NB, neuroborreliosis; *n*, number of study participants; NA, not applicable.

b Six active Lyme neuroborreliosis patients were also included as treated neuroborreliosis patients (>1 year after they had finished treatment for their Lyme neuroborreliosis disease episode).

c For all two-group comparisons with a significant difference, the Bonferroni correction was applied.

d Nine (27.3%) active Lyme neuroborreliosis patients did not complete the Lyme-specific questionnaire, so data on tick bite and/or EM were not present for them.

e One patient with erythema migrans did not recall a tick bite; all others did recall a tick bite.

f For active Lyme neuroborreliosis patients, instead of the self-reported complaints, we assessed the electronic patients files for clinical symptoms due to Lyme neuroborreliosis. Those symptoms are listed in Table 2.

g For one (2.7%) treated Lyme neuroborreliosis patient, two-tier serology testing was not done because of the lack of a serum sample.

h Two individuals with erythema migrans did not recall a tick bite and six individuals did not report an erythema migrans and were treated for an atypical skin rash (*n* = 4), flu-like symptoms after the tick bite (*n* = 1), or the presence of an engorged adult tick (*n* = 1).

i One (3.6%) individual who did not know when antibiotic treatment took place was excluded.

j All individuals with erythema migrans recalled a tick bite.

k Untreated healthy individuals versus active Lyme neuroborreliosis patients.

l Untreated healthy individuals versus all other groups.

m Treated healthy individuals versus all other groups (*P* ≤ 0.041); treated Lyme neuroborreliosis patients versus active Lyme neuroborreliosis patients (*P* = 0.033).

n Treated healthy individuals versus all other groups (*P* < 0.001); untreated healthy individuals versus both Lyme neuroborreliosis patient groups (*P* ≤ 0.015).

o Active Lyme neuroborreliosis patients versus all other groups.

p Active Lyme neuroborreliosis patients versus all other groups (*P* < 0.001); untreated healthy individuals versus treated Lyme neuroborreliosis patients (*P* = 0.025).

q Active Lyme neuroborreliosis patients versus all other groups.

The majority of the active Lyme neuroborreliosis patients, 25/33 (75.8%), were classified as definite Lyme neuroborreliosis patients and 8/33 (24.2%) of them as possible Lyme neuroborreliosis patients, because they lacked production of intrathecal antibody against Borrelia (Table 2). Only three patients had a positive antibody index (AI) based on a solitary IgM response, 12 patients had positive AIs for both IgM and IgG, and 10 patients had a positive AI based on a solitary IgG response (see Table S1 in the supplemental material).

**TABLE 2:** Clinical symptoms and case definitions based on the EFNS criteria (11) of treated and active Lyme neuroborreliosis patients in this study during their active disease period^*a*^

Active Lyme NB patients (*n* = 33)	Treated Lyme NB patients (*n* = 37)	Clinical symptoms			Median CSF leukocyte count during diagnosis (/μl) (IQR)	Intrathecal antibody production^*c*^	EFNS criterion	
		Radicular disease^*b*^	Cranial nerve paresis	Other			Possible Lyme NB	Definite Lyme NB
8^*d*^		x			56.5 (27.3–232.3)	x		x
2		x			131.8 (121.8–141.9)		x	
6			x		158.3 (82.7–254.5)	x		x
4			x		21.0 (17.3–67.5)		x	
2				x^*g*^	395.5 (304.8–486.3)	x		x
2				x^*g*^	94.2 (82.6–105.8)		x	
3^*e*^		x	x		80.0 (46.2–249.0)	x		x
1		x		x^*h*^	13.3	x		x
1^*f*^		x	x	x^*g*^	473.7	x		x
1			x	x^*g*^	377.3	x		x
3				x^*i*^	41.0 (29.0–116.8)	x		x
Median pleocytosis (IQR)					111.7 (21.0–243.5)			
Total (*n*; %)		15 (45.5)	15 (45.5)	10 (30.3)		25^*k*^ (75.8)	8 (24.2)	25 (75.8)
	15^*d*^	x			50.0 (32.5–105.8)	x		x
	2	x			<5	x	x	
	7		x		60.0 (44.0–83.5)	x		x
	2		x		<5	x	x	
	5^*e*^	x	x		88.0 (20.3–128.0)	x		x
	1	x	x		76.0		x	
	1^*f*^	x	x	x^*g*^	473.7	x		x
	1		x	x^*h*^	83.3	x		x
	2			x^*j*^	94.8 (74.3–115.4)	x		x
	1			x^*h*^	<5	x	x	
Median pleocytosis (IQR)					52.0 (22.2–105.9)			
Total (*n*; %)		24 (64.9)	17 (45.9)	5 (13.5)		36^*l*^^,^^*m*^ (97.3)	6 (16.2)	31 (83.8)
*P* value					0.071	0.010	0.402^*n*^	

a CSF, cerebrospinal fluid; EFNS, European Federation of Neurological Societies.

b Radicular disease was based on either radiculopathy, radiculitis, or radiculomyelitis.

c Detailed information regarding the antibody index for active Lyme neuroborreliosis patients can be found in Table S1.

d Four patients were included as active and treated Lyme neuroborreliosis patients.

e One patient was included as an active and treated Lyme neuroborreliosis patient.

f The patient was included as an active and treated Lyme neuroborreliosis patient.

g Patient diagnosed with meningitis.

h Patient diagnosed with peripheral neuropathy.

i One patient diagnosed with encephalitis, one patient diagnosed with a cerebrovascular accident, and one patient diagnosed with peripheral neuropathy.

j One patient diagnosed with meningitis and one patient diagnosed with peripheral neuropathy.

k Sixteen (48.5%) patients had a positive IgM antibody index, and 22 (66.7%) patients had a positive IgG antibody index. For one patient the IgM antibody index could not be determined.

l Seventeen (45.9%) patients had a positive IgM antibody index, and 36 (97.3%) had a positive IgG antibody index; see also Table S1.

m A significantly higher number of treated Lyme neuroborreliosis patients had intrathecal antibody production at the time they were diagnosed with active Lyme neuroborreliosis than in the active Lyme neuroborreliosis patient group.

n No difference was found in EFNS criteria between active Lyme neuroborreliosis patients and treated Lyme neuroborreliosis patients.

### (ii) Treated Lyme neuroborreliosis patients.

Thirty-seven Lyme neuroborreliosis patients were included at a median of 5.0 years (IQR, 2.5 to 7.3 years) after they had finished antibiotic therapy for LB (Table 1). The median age of the treated Lyme neuroborreliosis patients at inclusion was 59.3 years (IQR, 49.4 to 66.9 years) (Table 1). Antibiotic therapy consisted of intravenous ceftriaxone for 14 or 30 days; one patient switched to doxycycline (for 14 days) due to an allergic reaction to ceftriaxone. Most treated Lyme neuroborreliosis patients suffered from radicular disease (24/37 [64.9%]) and/or cranial nerve paresis (17/37 [45.9%]), which was similar to what was observed for the active Lyme neuroborreliosis patients (Table 2).

Thirty-one (83.8%) out of the 37 treated Lyme neuroborreliosis patients were, when they were diagnosed with active Lyme neuroborreliosis in the past, classified as definite Lyme neuroborreliosis patients and 6/37 (16.2%) as possible Lyme neuroborreliosis patients, of whom the majority did not have pleocytosis (5/6 [83.3%]) (Table 2). This was in contrast with the active Lyme neuroborreliosis patients, who were, when diagnosed with active Lyme neuroborreliosis, more often classified as possible Lyme neuroborreliosis patients, because of the absence of intrathecally produced Borrelia-specific antibodies (*P* = 0.010) (Table 2). A total of 36 (97.3%) of the 37 treated Lyme neuroborreliosis patients had a positive AI for IgG, of whom 17 (47.2%) also had a positive AI for IgM (data not shown). Interestingly, 23/37 (62.2%) of the treated Lyme neuroborreliosis patients still reported complaints when they were included in this study (Table 1). These self-reported complaints included neuropathic complaints, cognitive complaints, fatigue, myalgias, paraesthesias, and/or malaise. A total of six treated Lyme neuroborreliosis patients had also been included as active Lyme neuroborreliosis patients at the time they were diagnosed with active Lyme neuroborreliosis; the median time between the end of antibiotic treatment for Lyme neuroborreliosis and inclusion in this study as a treated Lyme neuroborreliosis patient was 2.3 years (IQR, 1.6 to 2.7 years).

### (iii) Healthy individuals.

One hundred seventy-three healthy individuals were included; their median age at inclusion was 42.2 years (IQR, 27.5 to 53.2 years). Twenty-eight (16.2%) out of these individuals reported antibiotic therapy for LB in the past (median, 5 years ago; IQR, 2 to 7 years), and they were classified separately as treated healthy individuals (Table 1). Most treated healthy individuals reported antibiotic treatment for EM (22/28 [78.6%]); the six remaining individuals were treated for an atypical skin rash (*n* = 4), flu-like symptoms after the tick bite (*n* = 1), or the presence of an engorged adult tick (*n* = 1). The remaining 145 (83.8%) healthy individuals were classified as untreated healthy individuals. The median age of the treated healthy individuals was 52.7 years (IQR, 38.1 to 57.5 years). In this group, the percentage of tick bites was higher than in all other groups (*P* ≤ 0.041). Comparison of the four study groups showed that the percentage of EM was also highest among treated healthy individuals (22/28 [78.6%]) (*P* ≤ 0.001) (Table 1). The untreated healthy individuals were younger than the other three groups (median, 41.0 years; IQR, 27.0 to 51.7 years) (*P* ≤ 0.029), and 87/145 (60.0%) recalled a tick bite; four of them also mentioned an EM. The percentage of reported EM within this group was lower than for all other groups (*P* ≤ 0.015) (Table 1).

### Two-tier serology results.

Serology testing showed that most of the active Lyme neuroborreliosis patients were seropositive (30/33 [90.9%]) (Table 1). Twenty-eight (84.8%) of the 33 seropositive patients had IgG antibodies; 16/33 (48.5%) also had IgM antibodies (Table 1). Only two active Lyme neuroborreliosis patients had a positive serology result based on a solitary IgM response. Both IgM and IgG were more often found among active Lyme neuroborreliosis patients than among the other three groups (*P* < 0.001 for both) (Table 1).

For 36/37 treated Lyme neuroborreliosis patients, a serum sample was available for serology; 6/36 (16.7%) had a positive result (Table 1). For all six cases, the result was based on an IgG response; three of them also had an IgM response (Table 1). Interestingly, no difference was found in serology among treated Lyme neuroborreliosis patients with and without complaints (*P* = 1.000; data not shown). Only 4 (17.4%) out of the 23 treated Lyme neuroborreliosis patients with complaints had Borrelia-specific antibodies, and 2 (15.4%) out of the 13 treated Lyme neuroborreliosis patients without complaints were seropositive (the serum of one patient without complaints was missing). The two-tier serology of the six patients that were included both as an active Lyme neuroborreliosis patient and later as a treated Lyme neuroborreliosis patient showed that four (66.7%) out of the six patients reverted from seropositive to seronegative. These six patients all had Lyme neuroborreliosis-specific symptoms at the time of their diagnosis with active disease (all had radicular disease; two had facial nerve paresis as well, of whom one also had meningitis). These Lyme neuroborreliosis-specific symptoms had all disappeared at the time they were included in the study as a treated Lyme neuroborreliosis patient. Two (33.3%) of them did not report any complaints at all, but four (66.7%) reported nonspecific symptoms, such as fatigue (*n* = 2), loss of focus and/or amnesia (*n* = 2), loss of strength (*n* = 1), early-onset rheumatoid arthritis (*n* = 1), urinary problems (*n* = 1), and arrhythmia (*n* = 1).

Among the 173 healthy individuals, a total of 23 (13.3%) had Borrelia-specific serum antibodies. Positive serology results were found among both treated and untreated healthy individuals (5/28 [17.9%] and 18/145 [12.4%], respectively) (Table 1). Among treated healthy individuals, positive serology results were based on either an IgG response (3/5 [60.0%]) or an IgM response (2/5 [40.0%]). Among untreated healthy individuals, all 18 positive serology results were based on an IgG response; only 1 (5.6%) of them also had an IgM response (Table 1). All 23 healthy individuals with a positive serology result were invited to consult an infectious diseases specialist, and 17 (73.9%) of them did indeed visit the specialist. None of them had any signs or symptoms suggesting a current or recent (symptomatic) LB.

### Performance of the Borrelia ELISpot assay with different Borrelia antigens.

Analysis of the final spot counts of both operators showed that one operator systematically had higher spot counts. The correlation between both operators, however, was very high (*r_s_*, 0.913; *P* < 0.001).

All 243 study participants were tested with B. burgdorferi B31 whole-cell lysate; a subset of study participants was also tested with the other Borrelia antigens (Table 3). In general, stimulation with Osp mix resulted in fewer IFN-γ-secreting T cells/2.5 × 10^5^ PBMCs than stimulation with B. burgdorferi B31 (*P* < 0.001 [all study participants] and *P* ≤ 0.028 [within the study groups]) (Table 3). A similar trend was seen for the different recombinant antigens compared to B. burgdorferi B31 (*P* < 0.001 [all study participants] and *P* ≤ 0.020 [within the study groups]) (Table 3). Interestingly, only for active Lyme neuroborreliosis patients was an association found between the number of Osp mix-specific IFN-γ-secreting T cells/2.5 × 10^5^ PBMCs and the number of B. burgdorferi B31-specific IFN-γ-secreting T cells/2.5 × 10^5^ PBMCs (*r_s_*, 0.723; *P* < 0.001; *n* = 21) (data not shown). Since the B. burgdorferi B31 lysate resulted in the highest number of IFN-γ-secreting T cells/2.5 × 10^5^ PBMCs, those results were used in all further (statistical) analyses.

**TABLE 3:** Various Borrelia antigens used for stimulating T cells in the Borrelia ELISpot assay

Study groups	Parameter	B. burgdorferi B31	Osp mix^*b*^	Recombinant antigens					B. burgdorferi B31 vs Osp mix^*b*^ *P* value	B. burgdorferi B31 vs recombinant antigen *P* value	
				B. burgdorferi p18 PKa	B. afzelii p18 PKo	B. garinii p18 PBi	B. afzelii p39 PKo	B. garinii p58 PBi		Overall	2-group
All study participants	*n*	243	95	119	118	118	118	118			
	%	100	39.1	49.0	48.6	48.6	48.6	48.6			
	Borrelia ELISpot result^*a*^	3.0	1.0	0.0	0.0	0.0	0.0	1.0	<0.001^*e*^	<0.001	≤0.047^*f*^
	IQR	1.0–6.5	0.0–2.5	0.0–1.0	0.0–1.1	0.0–1.0	0.0–2.0	0.0–2.6			
Active Lyme NB patients	*n*	33	21	16	16	16	16	16			
	%	100	63.6	48.5	48.5	48.5	48.5	48.5			
	Borrelia ELISpot result^*a*^	6.0	1.0	0.0	0.0	0.0	0.0	1.5	0.001^*e*^	<0.001	≤0.020^*g*^
	IQR	0.5–14.0	0.0–4.5	0.0–0.8	0.0	0.0–1.8	0.0–1.4	1.0–4.9			
Treated Lyme NB patients	*n*	37	13	22	22	22	22	22			
	%	100	35.1	59.5	59.5	59.5	59.5	59.5			
	Borrelia ELISpot result^*a*^	4.5	0.0	0.0	0.0	0.5	0.0	2.8	0.021^*e*^	<0.001	<0.001^*h*^
	IQR	2.0–18.6	0.0–5.0	0.0–1.0	0.0–2.6	0.0–2.0	0.0–2.1	0.0–5.3			
Treated healthy individuals	*n*	28	12	14	14	14	14	14			
	%	100	42.9	50.0	50.0	50.0	50.0	50.0			
	Borrelia ELISpot result^*a*^	7.4	2.0	0.0	1.0	0.0	1.0	1.0	0.028^*e*^	<0.001	≤0.009^*i*^
	IQR	2.3–14.9	0.3–6.0	0.0–1.0	0.0–2.3	0.0–2.0	0.0–6.0	0.0–3.3			
Untreated healthy individuals	*n*	145	49	67	66	66	66	66			
	%	100	33.8	46.2	45.5	45.5	45.5	45.5			
	Borrelia ELISpot result^*a*^	2	1	0	0	0	0	0	0.017^*e*^	<0.001	<0.001^*j*^
	IQR	0.5–3.9	0–1.5	0–1.0	0–1.0	0–1.0	0–1.6	0–1.0			
*P* value (overall)		<0.001	0.227	0.735	0.311	0.943	0.219	0.002			
*P* value (2-group)		≤0.016^*c*^	NC^*k*^	NC	NC	NC	NC	≤0.004^*d*^			

a The Borrelia ELISpot assay result reflects the median count of the number of activated T cells by the corresponding antigen used/2.5 × 10^5^ peripheral blood mononuclear cells.

b The Osp mix consists of a pool of 9-mer to 11-mer peptides of OspA (B. burgdorferi, B. afzelii, and B. garinii), native OspC (B. afzelii), and recombinant p18.

c Untreated healthy individuals had significantly lower numbers of B. burgdorferi B31-specific IFN-γ-secreting T cells/2.5 × 10^5^ peripheral blood mononuclear cells than both treated groups (*P* < 0.001) and active Lyme neuroborreliosis patients (*P* = 0.016) (see also Fig. 1).

d Untreated healthy individuals had significantly lower numbers of B. garinii p58 PBi-specific IFN-γ-secreting T cells/2.5 × 10^5^ peripheral blood mononuclear cells than active Lyme neuroborreliosis patients.

e Among all study participants, stimulation with Osp mix resulted in significantly lower numbers of activated T cells/2.5 × 10^5^ peripheral blood mononuclear cells than stimulation with B. burgdorferi B31 (*P* < 0.001). This was also found among the four study groups separately (*P* = 0.001 to 0.028).

f For all five recombinant antigens, stimulation resulted in significantly lower numbers of activated T cells/2.5 × 10^5^ peripheral blood mononuclear cells than stimulation with B. burgdorferi B31 (*P* < 0.001). Stimulation with B. burgdorferi B31 PKa also resulted in a lower number of activated T cells/2.5 × 10^5^ peripheral blood mononuclear cells than stimulation with B. garinii p58 PBi (*P* = 0.047).

g For two recombinant antigens, B. burgdorferi p18 PKa and B. afzelii p18 PKo, stimulation resulted in significantly lower numbers of activated T cells/2.5 × 10^5^ peripheral blood mononuclear cells than stimulation with B. burgdorferi B31 (*P* < 0.020) or stimulation with B. garinii p58 PBi (*P* < 0.017).

h For four recombinant antigens, stimulation resulted in significantly lower numbers of activated T cells/2.5 × 10^5^ peripheral blood mononuclear cells than stimulation with B. burgdorferi B31; the exception was B. garinii p58 PBi.

i For four recombinant antigens, stimulation resulted in significantly lower numbers of activated T cells/2.5 × 10^5^ peripheral blood mononuclear cells than stimulation with B. burgdorferi B31; the exception was B. afzelii p39 PKo.

j For all recombinant antigens, stimulation resulted in significantly lower numbers of activated T cells/2.5 × 10^5^ peripheral blood mononuclear cells than stimulation with B. burgdorferi B31.

k NC, not calculated.

### Borrelia ELISpot assay results by study group and self-reported complaints.

No significant difference was found in the number of B. burgdorferi B31-specific IFN-γ-secreting T cells/2.5 × 10^5^ PBMCs between the following three groups: active Lyme neuroborreliosis patients (median, 6.0; IQR, 0.5 to 14.0), treated Lyme neuroborreliosis patients (median, 4.5; IQR, 2.0 to 18.6), and treated healthy individuals (median, 7.4; IQR, 2.3 to 14.9) (*P* ≥ 1.000) (Fig. 1). However, these three groups had higher numbers of B. burgdorferi B31-specific IFN-γ-secreting T cells/2.5 × 10^5^ PBMCs than untreated healthy individuals (median, 2.0; IQR, 0.5 to 3.9) (*P* ≤ 0.016) (Table 3).

**FIG 1:**
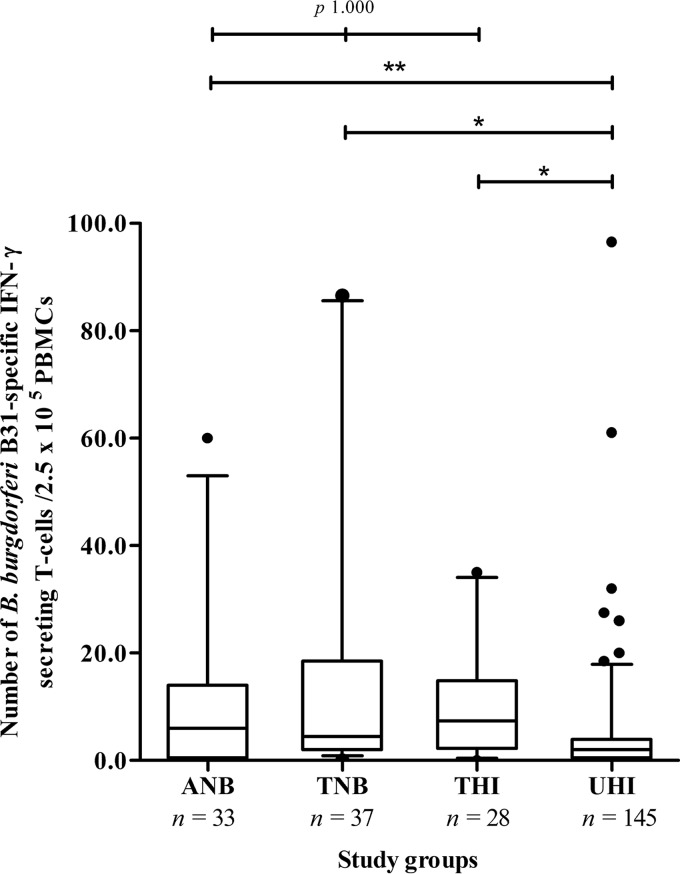
B. burgdorferi B31-specific T-cell activation among active Lyme neuroborreliosis patients, treated Lyme neuroborreliosis patients, treated healthy individuals, and untreated healthy individuals. ANB, active Lyme neuroborreliosis patients; TNB, treated Lyme neuroborreliosis patients; THI, treated healthy individuals; UHI, untreated healthy individuals; *n*, number of study participants. *, significant difference based on a *P* value of <0.001; **, significant difference based on a *P* value of 0.016.

More than 60% of the treated Lyme neuroborreliosis patients reported one or more symptoms in the Lyme-specific questionnaire (Table 1); however, no correlation was found between these self-reported symptoms and the number of B. burgdorferi B31-specific IFN-γ-secreting T cells/2.5 × 10^5^ PBMCs (*r_s_*, 0.200; *P* = 0.235). The reactivity found among treated healthy individuals also could not be linked to symptomatic disease, since none of the healthy individuals reported any complaints.

### Diagnostic performance of the Borrelia ELISpot assay.

**FIG 2:**
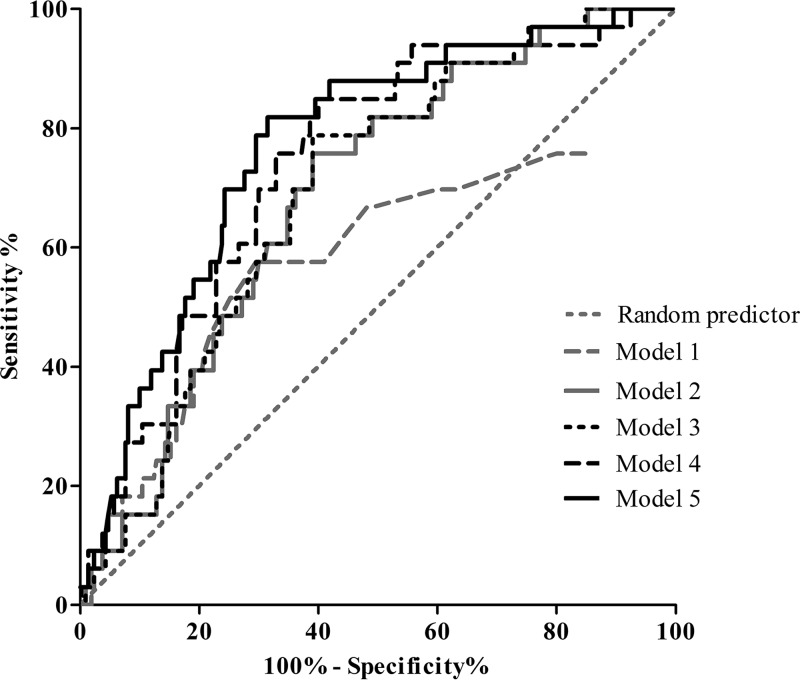
Receiver operating characteristic (ROC) curve of the Borrelia ELISpot assay results and selected logistic regression models that improved the diagnostic performance of the Borrelia ELISpot assay used in this study. The ROC curve of model 1 is based on the number of B. burgdorferi B31-specific IFN-γ-secreting T cells/2.5 × 10^5^ PBMCs (the Borrelia ELISpot assay). Model 2 is based solely on the risk factors tick bite and age and thus is without the addition of the Borrelia ELISpot assay results. Model 3 is based on model 2, with the addition of the Borrelia ELISpot assay results. Model 4 is based on all risk factors analyzed in this study (i.e., sex, tick bite, EM, and age, in addition to the Borrelia ELISpot assay results), and model 5 is based on model 4 with the addition of the interaction term “age by Borrelia ELISpot” (see also Table S2).

To assess the diagnostic performance of the Borrelia ELISpot assay for detecting active Lyme neuroborreliosis, we used a logistic regression model. In the first model, only the results of the Borrelia ELISpot assay were used. The outcome of the model was used to create a receiver operating characteristic (ROC) curve. Unfortunately, the area under the curve (AUC) found was only slightly better than a random predictor (model 1; AUC, 0.591) (Fig. 2) and the model did not fit the data (*P* = 0.026) (see Table S2 in the supplemental material).

To determine the (added) value of various risk factors, we also investigated other logistic regression models. The following risk factors were assessed: sex, tick bite, EM, and age (see Table S2). Interestingly, when a model was created for which only the risk factors tick bite and age were included, and thus without the results of the Borrelia ELISpot assay, a better AUC was achieved (model 2; AUC, 0.689) (Fig. 2; see also Table S2). Addition of the Borrelia ELISpot assay results to the risk factors of model 2 only minimally increased the AUC (model 3; AUC, 0.694) (Fig. 2; see also Table S2). When all risk factors were included in the model, an AUC of 0.741 was found (model 4). Taking into account all possible interaction effects, only “age by Borrelia ELISpot” was significant (*P* = 0.018), and adding this to model 4 resulted in an AUC of 0.769 (model 5) (Fig. 2; see also Table S2). In the last model, the absence of a tick bite increased the odds of being an active Lyme neuroborreliosis patient (odds ratio [OR], 2.938; *P* = 0.029). The contribution of the Borrelia ELISpot assay result (OR, 1.218; *P* = 0.010), age (OR, 1.061; *P* = 0.001), and the interaction term “age by Borrelia ELISpot” (OR, 0.996; *P* = 0.018) was minimal in this model (see Table S2).

### Borrelia ELISpot assay versus two-tier serology.

In general, seropositive cases had a higher number of B. burgdorferi B31-specific IFN-γ-secreting T cells/2.5 × 10^5^ PBMCs (median, 5.0; IQR, 1.5 to 14.0) than seronegative cases (median, 2.0; IQR, 1.0 to 5.0) (*P* = 0.005) (Table 4). When the four study groups were analyzed separately, no significant difference was found in the number of B. burgdorferi B31-specific IFN-γ-secreting T cells/2.5 × 10^5^ PBMCs between seropositive and seronegative cases (*P* = 0.070 to 1.000) (Table 4). Interestingly, among seronegative study participants, less B. burgdorferi B31-specific T-cell activity was found among untreated healthy individuals than in both treated groups (*P* ≤ 0.001) (data not shown); no difference was found among the seropositive study participants between the four study groups (*P* = 0.216) (data not shown). Analysis of the C6 ELISA index scores, which are semiquantitative, showed an association between the level of the C6 ELISA index scores and the number of Borrelia-specific T cells (*r_s_*, 0.187; *P* = 0.004); however, no association was found within any of the four groups (data not shown).

**TABLE 4:** Overview of the number of B. burgdorferi B31-specific IFN-γ-secreting T cells among study participants with and without Borrelia-specific serum antibodies

Group	Serology result	*n*	No. of B. burgdorferi B31-specific IFN-γ-secreting T cells/2.5 × 10^5^ PBMCs		*P* value
			Median	IQR	
All combined	−	183	2.0	1.0–5.0	0.005^*b*^
	+	59	5.0	1.5–14.0	
Active Lyme NB patients	−	3	5.0	2.5–19.5	1.000
	+	30	6.0	0.8–13.5	
Treated Lyme NB patients^*a*^	−	30	5.5	2.0–18.1	0.664
	+	6	4.3	3.5–36.6	
Treated healthy individuals	−	23	6.0	1.5–14.0	0.121
	+	5	15.0	4.0–34.0	
Untreated healthy individuals	−	127	1.5	0.5–3.8	0.070
	+	18	3.0	1.0–6.3	

a For one treated Lyme neuroborreliosis patient, two-tier serology testing was not done because of the lack of a serum sample.

b Seronegative study participants had significantly lower numbers of B. burgdorferi B31-specific IFN-γ-secreting T cells/2.5 × 10^5^ peripheral blood mononuclear cells than seropositive study participants.

### Borrelia ELISpot assay versus antibody index for active Lyme neuroborreliosis patients.

Because of the prerequisite of a CSF sample to determine the antibody index, AIs were determined only for Lyme neuroborreliosis patients at the time of diagnosis and thus were lacking for the healthy individuals. Only for active cases were the AI and Borrelia ELISpot assay results from samples from the same time period available and thus comparable and could be used in subsequent analyses. No difference was found among active Lyme neuroborreliosis patients when the numbers of B. burgdorferi B31-specific IFN-γ-secreting T cells/2.5 × 10^5^ PBMCs were compared between AI-positive and AI-negative cases (*P* = 0.550) (see Table S1). Similarly, no difference was found among the active patients when the numbers of B. burgdorferi B31-specific IFN-γ-secreting T cells/2.5 × 10^5^ PBMCs were compared between negative and positive IgM AI results or between negative and positive IgG AI results (*P* = 0.081 and 0.336, respectively) (see Table S1). The lack of an association between the number of B. burgdorferi B31-specific IFN-γ-secreting T cells/2.5 × 10^5^ PBMCs and the level of AI scores of both IgM and IgG was confirmed using Spearman's correlation coefficient (*r_s_* = 0.109 and *P* = 0.575 for IgM and *r_s_* = −0.054 and *P* = 0.764 for IgG). We did, however, find a negative correlation between the level of the AI score for IgM and the number of B. burgdorferi B31-specific IFN-γ-secreting T cells/2.5 × 10^5^ PBMCs when only those active patients who had a positive AI for IgM were analyzed (*n* = 12; *r_s_*, −0.694; *P* = 0.012). No such association was found for IgG (data not shown).

## DISCUSSION

In this study, we used well-defined patient populations and healthy controls to evaluate the utility of the Borrelia ELISpot assay. We found that the number of B. burgdorferi B31-specific IFN-γ-secreting T cells/2.5 × 10^5^ PBMCs in peripheral blood was significantly elevated in active Lyme neuroborreliosis patients, treated Lyme neuroborreliosis patients, and healthy individuals treated for early manifestations of LB in the past compared to untreated healthy individuals (*P* ≤ 0.016). Thus, positive Borrelia ELISpot assay results are, in general, associated with exposure and/or (past) infection with B. burgdorferi sensu lato. The diagnostic performance of the Borrelia ELISpot assay for the detection of active disease was determined by calculation of the ROC curve, which resulted in an AUC of 0.591, suggesting that this assay is unsuitable for the diagnosis of active Lyme neuroborreliosis.

To diagnose Lyme neuroborreliosis, laboratories often rely upon the detection of intrathecally produced Borrelia-specific antibodies. The Borrelia ELISpot assay, however, did not outperform the AI assay, as the Borrelia ELISpot assay results among active Lyme neuroborreliosis patients did not differ between AI-positive and AI-negative patients (*P* = 0.550). We did, however, find a negative correlation among positive AI scores for IgM and the number of B. burgdorferi B31-specific IFN-γ-secreting T cells/2.5 × 10^5^ PBMCs in peripheral blood (*r_s_*, −0.694; *P* = 0.012). This is in line with the results found by Dattwyler et al. (36, 37), who showed that a Borrelia-specific T-cell response precedes the development of a measurable antibody response.

In this study, we found that whole-cell lysates of B. burgdorferi B31 yielded more activated T cells when used to stimulate the PBMCs than when various recombinant antigens were used for PBMC stimulation. This could be explained by the higher number of antigens present in whole-cell lysates and, hence, more antigenic determinants that can elicit an immune response than the (limited) number of antigenic determinants present among the recombinant antigens used. von Baehr et al. (38) also reported that a whole-cell lysate stimulated PBMCs better than recombinant antigens. We cannot, however, exclude the possibility that the amount of recombinant antigens we used was too low.

We found Borrelia ELISpot assay reactivity among treated Lyme neuroborreliosis patients and treated healthy individuals, but this could not be linked to symptoms, although more than 60% of the treated Lyme neuroborreliosis patients in this study still reported (nonspecific) symptoms. Similar percentages have been found in other studies (39–41). The nonspecific symptoms reported among treated Lyme neuroborreliosis patients could not be linked to the Borrelia ELISpot assay results. The Borrelia ELISpot assay reactivity among the treated healthy individuals could also not be linked to complaints, as these individuals were included in the study only when they reported having no complaints at all. Therefore, we conclude that the Borrelia ELISpot assay reactivity among both treated groups is most likely explained by a previous, cured LB.

Borrelia ELISpot assay reactivity was also found among untreated healthy individuals. Ekerfelt et al. (42) also found elevated numbers of Borrelia-specific IFN-γ-secreting T cells in both clinical LB cases and asymptomatic (seropositive) controls after stimulation with an outer surface-enriched fraction of B. afzelii. In our study, the number of B. burgdorferi B31-specific IFN-γ-secreting T cells/2.5 × 10^5^ PBMCs did not differ between seropositive and seronegative untreated healthy individuals (*P* = 0.070). This could be explained by the low number of seropositive cases (18 seropositive cases versus 127 seronegative cases), although Dattwyler et al. (43) did report an increased T-cell proliferative response to whole-cell B. burgdorferi among active LB patients who did not have Borrelia-specific antibodies. Borrelia ELISpot assay reactivity among untreated, seronegative healthy individuals could also be explained by the choice of the antigen. It is known that the use of whole-cell lysates increases the chance of cross-reactivity, which could lead to false-positive results. The B. burgdorferi B31 lysate used contains antigens such as flagellin, which shows high homology with antigens from Treponema pallidum or bacteria of the genus Leptospira. To investigate for possible cross-reactivity, we also tested the blood of some patients with active neurosyphilis (*n* = 2) and active leptospirosis (*n* = 2), and a strong ELISpot assay reactivity against B. burgdorferi B31 was found for one leptospirosis case, but the patients with neurosyphilis did not show Borrelia ELISpot assay reactivity (data not shown). Lipopolysaccharides (LPS) present in whole-cell lysates could also potentially stimulate the T cells, although Janský et al. (18) showed that LPS from Escherichia coli did not result in elevated IFN-γ levels and that Borrelia lysates did. Other studies showed the production of IFN-γ by NK cells after stimulation with LPS (44, 45); however, we tried to correct for this phenomenon by adjusting the ELISpot assay reader settings in which we omitted small- and low-intensity spots (35, 46).

Apart from the possible false-positive results, false-negative results were also found, as samples from some active Lyme neuroborreliosis patients did not show Borrelia ELISpot assay reactivity. This could be explained by the type of species used to stimulate the T cells, since B. garinii and B. bavariensis have been linked to Lyme neuroborreliosis more often than B. burgdorferi, and B. afzelii has been isolated from CSF of Lyme neuroborreliosis patients as well (47, 48). Since all aforementioned Borrelia species are closely related and share many antigens, they will most likely be cross-reactive when used in the Borrelia ELISpot assay. Therefore, we decided to test the B. burgdorferi B31 lysate, supported by the results of von Baehr et al. (38) and Nordberg et al. (49). von Baehr et al. tested three different Borrelia species in a lymphocyte transformation test and did not find any differences between these species. Nordberg et al. used B. garinii as a stimulating agent in an ELISpot assay among Lyme neuroborreliosis patients and obtained results which were comparable with the results of the B. burgdorferi B31 ELISpot assay we tested. The relatively low numbers of B. burgdorferi B31-specific IFN-γ-secreting T cells/2.5 × 10^5^ PBMCs among active Lyme neuroborreliosis patients in our study could also be explained by the compartmentalization of T cells to the CSF. Several studies have shown that patients with neurological LB had less T-cell reactivity against Borrelia in peripheral blood than other manifestations of LB (22, 23). Analysis of the T-cell response in CSF and blood in a subset of patients who had neurological LB also showed a higher number of Borrelia-specific IFN-γ-secreting T cells in CSF than in blood (22). Still, analysis of CSF did not result in a better diagnostic performance, as has been shown by Nordberg et al. (49), who found a sensitivity of 36% and a specificity of 82% using five spots.

In our study, we included only active Lyme neuroborreliosis patients as defined by the EFNS criteria (11). These criteria are clear and easy to apply. Most active and treated Lyme neuroborreliosis patients in this study were deemed to have definite Lyme neuroborreliosis at the time of diagnosis (75.8% and 83.8%, respectively), and therefore, we feel confident that we were dealing with true LB cases. We used the active Lyme neuroborreliosis patients as a proxy for active LB; however, we realize that it is difficult to extrapolate the results found in this study to other manifestations of LB. Future studies should therefore include patients with other manifestations of LB as well. Our research group has started to include Lyme arthritis cases since the beginning of 2015 and intends to include other LB manifestations, such as EM, Lyme lymphocytoma, or ACA, in 2018 as well.

This study had various limitations. A difference was found in sex and age between the four study groups. Patients with active Lyme neuroborreliosis were more often male and were older than untreated healthy individuals, which is most likely explained by the way of recruitment, as most healthy individuals were recruited in our hospital, increasing the likelihood of inclusion of more (young) females. The results of the logistic regression model, however, did not show any significant attribution for sex, and the contribution of age was minimal (OR, 1.061; *P* = 0.001).

The way of recruitment also led to the inclusion of increased numbers of healthy individuals with a past tick bite and/or EM. The results of the logistic regression model indeed showed that the absence of a tick bite could aid in diagnosing active Lyme neuroborreliosis. Although most likely biased, there could be some logic in the contribution of this risk factor in developing active Lyme neuroborreliosis, because not noticing a tick bite could increase the chance of developing disseminated LB. One would expect individuals who did notice a tick bite to be more alert for development of any symptoms suggesting LB, and those individuals would consequently seek medical advice if they developed such symptoms. They are, therefore, less likely to develop disseminated LB.

The way of recruitment of the three control groups, which included treated Lyme neuroborreliosis patients as well as healthy individuals with a previously treated early manifestation of LB and/or an increased risk of contact with the Borrelia bacterium, resulted in increased Borrelia ELISpot assay reactivity and thus lower specificity. Therefore, further studies should include cohorts with lower prevalences, as well as other (cross-reacting) diseases to better assess the specificity of the Borrelia ELISpot assay.

Unfortunately, not much is known about the T-cell dynamics after treatment, and controversial data have been published regarding this subject (23, 50, 51). Therefore, this needs to be further elucidated, and we are currently monitoring the active Lyme neuroborreliosis patients both serologically and immunologically (through Borrelia ELISpot assay) at different time points up to 2 years after inclusion. This way we hope to get more information regarding the T-cell dynamics.

Finally, a total of six patients were included twice in this study, both as active Lyme neuroborreliosis patients and, at a later time point (≥1.6 years later), as treated Lyme neuroborreliosis patients. As 66.7% turned seronegative and Lyme-specific symptoms at the time of active disease had disappeared and the individuals showed either nonspecific symptoms or a complete recovery, we do not believe that this created a bias.

In conclusion, the Borrelia ELISpot assay used in this study, measuring the number of B. burgdorferi B31-specific IFN-γ-secreting T cells, cannot be used for the diagnosis of active Lyme neuroborreliosis.

## Supplementary Material

Supplemental material
