# Supplementary material for: An Enzyme-Linked Immunosorbent Spot Assay Measuring Borrelia burgdorferi B31-Specific Interferon Gamma-Secreting T Cells Cannot Discriminate Active Lyme Neuroborreliosis from Past Lyme Borreliosis: a Prospective Study in the Netherlands
Source: J Clin Microbiol. 2018 Mar 26;56(4):e01695-17. doi: 10.1128/JCM.01695-17 (PMC5869815; doi:10.1128/JCM.01695-17)
Supplement: Supplemental material [file supp_56_4_e01695-17__index.html]

An Enzyme-Linked Immunosorbent Spot Assay Measuring Borrelia burgdorferi B31-Specific Interferon Gamma-Secreting T Cells Cannot Discriminate Active Lyme Neuroborreliosis from Past Lyme Borreliosis: a Prospective Study in the Netherlands — Supplemental material 

# An Enzyme-Linked Immunosorbent Spot Assay Measuring Borrelia burgdorferi B31-Specific Interferon Gamma-Secreting T Cells Cannot Discriminate Active Lyme Neuroborreliosis from Past Lyme Borreliosis: a Prospective Study in the Netherlands

## Supplemental material

- Supplemental file 1 -

  Tables S1 (Antibody index and *Borrelia* ELISpot assay results among the active Lyme neuroborreliosis patients in this study) and S2 (Various logistic regression models assessing risk factors which could contribute to the diagnostic performance of the *Borrelia* ELISpot assay used in this study)

  PDF, 43K
