# Supplementary material for: An Enzyme-Linked Immunosorbent Spot Assay Measuring Borrelia burgdorferi B31-Specific Interferon Gamma-Secreting T Cells Cannot Discriminate Active Lyme Neuroborreliosis from Past Lyme Borreliosis: a Prospective Study in the Netherlands
Source: J Clin Microbiol. 2018 Mar 26;56(4):e01695-17. doi: 10.1128/JCM.01695-17 (PMC5869815; doi:10.1128/JCM.01695-17)
Supplement: Supplemental material [file JCM.01695-17_zjm999095884s1.pdf]

Table S1. Antibody index and *Borrelia* ELISpot assay results among the active Lyme neuroborreliosis patients in this study

Test	Active Lyme neuroborreliosis patients (*n* = 33)		
	**neg**	**pos**	***P* value**
**Ig total AI result (*n*; %)**	8 (24.2)	25 (75.8)	
Borrelia ELISpot[b] (median; IQR)	8.0 (2.3-21.8)	6.0 (0.0-10.8)	0.550
**IgM AI result (*n*; %)**	17 (51.5)	16 (48.5)	
AI score[a] (median; IQR)	<0.3	3.9 (3.2-15.6)[c]	
Borrelia ELISpot[b] (median; IQR)	2.0 (0.0-14.0)	6.8 (5.0-15.8)	0.081
**IgG AI result (*n*; %)**	11 (33.3)	22 (66.7)	
AI score[a] (median; IQR)	<0.3	11.1 (2.9-27.1)	
Borrelia ELISpot[b] (median; IQR)	6.0 (3.0-24.0)	5.5 (0.0-10.1)	0.336

Ig: immunoglobulin; AI: antibody index; IQR: interquartile range

a) An antibody index score ≥0.3 is regarded as positive;
b) The *Borrelia* ELISpot assay results are based on the number of *B. burgdorferi* B31-specific IFN-γ-secreting T cells/2.5 x 10^5 peripheral blood mononuclear cells;
c) For four active Lyme neuroborreliosis patients, the antibody index scores were lacking; however, in the electronic patient files the final result for all four were recorded as positive.

Table S2. Various logistic regression models assessing risk factors which could contribute to the diagnostic performance of the *Borrelia* ELISpot assay used in this study

Covariates logistic regression	Logistic regression models				
	**1** *n* = 243	**2** *n* = 234[c]	**3** *n* = 234[c]	**4** *n* = 234[c]	**5[d]** *n* = 234[c]
**Factors (*P* values)**					
Sex (male)				0.071	0.106
Tick bite (no)		0.055	0.054	0.037	0.029
EM (no)				0.964	0.593
**Continues variables (*P* values)**					
Age		0.012	0.013	0.014	0.001
Borrelia ELISpot[a]	0.126		0.841	0.872	0.010
**Interaction term (*p* values)**					
Age by *Borrelia* ELISpot[a]					0.018
**AUC**	0.591	0.689	0.694	0.741	0.769
**Model fit[b]**	0.026	0.733	0.726	0.519	0.809
Sex (male)				2.282 (0.931-5.595)	2.117 (0.853-5.256)
Tick bite (no)		2.334 (0.981-5.555)	2.351 (0.985-5.612)	2.690 (1.062-6.815)	2.938 (1.119-7.710)
EM (no)				0.972 (0.281-3.365)	0.706 (0.197-2.529)
Age		1.039 (1.008-1.071)	1.039 (1.008-1.071)	1.038 (1.007-1.069)	1.061 (1.024-1.101)
Borrelia ELISpot[a]	1.016 (0.995-1.037)		1.003 (0.975-1.032)	1.002 (0.974-1.031)	1.218 (1.049-1.415)
Age by *Borrelia* ELISpot[a]					0.996 (0.993-0.999)

EM: erythema migrans; AUC: area under the curve; *n*: number of study participants; C.I.: confidence interval; OR: odds ratio

The logistic regression model calculates the added value of various risk factors for determining active Lyme neuroborreliosis; only a few (combinations of) risk factors are shown.

a) The *Borrelia* ELISpot assay results are based on the number of *B. burgdorferi* B31-specific IFN-γ-secreting T cells/2.5 x 10^5 peripheral blood mononuclear cells;
b) The Hosmer-Lemeshow goodness of fit test was used to assess whether the model fitted the data;
c) Nine active Lyme neuroborreliosis patients did not complete the Lyme-specific questionnaire, so data on tick bite and erythema migrans was not available. Therefore, models including one or both of these risk factors were based on a total of 234 cases instead of 243;
d) The randomForest method using the Gini coefficient (R studio, version 1.1.383, 2009-2017 RStudio, Inc.) was also performed and confirmed that model 5 fitted the data best (data not shown).
